# Artificial intelligence in clinical medicine: a state-of-the-art overview of systematic reviews with methodological recommendations for improved reporting

**DOI:** 10.3389/fdgth.2025.1550731

**Published:** 2025-03-05

**Authors:** Giovanni Morone, Luigi De Angelis, Alex Martino Cinnera, Riccardo Carbonetti, Alessio Bisirri, Irene Ciancarelli, Marco Iosa, Stefano Negrini, Carlotte Kiekens, Francesco Negrini

**Affiliations:** ^1^ Department of Life, Health and Environmental Sciences, University of L'Aquila, L’Aquila, Italy; ^2^ San Raffaele Institute of Sulmona, Sulmona, Italy; ^3^ Department of Translational Research and New Technologies in Medicine and Surgery, University of Pisa, Pisa, Italy; ^4^ Italian Society of Artificial Intelligence in Medicine (SIIAM, Società Italiana Intelligenza Artificiale in Medicina), Rome, Italy; ^5^ Scientific Institute for Research, Hospitalisation and Health Care IRCCS Santa Lucia Foundation, Rome, Italy; ^6^ Clinical Area of Neuroscience and Neurorehabilitation, Neurofunctional Rehabilitation Unit, IRCCS “Bambino Gesù” Children’s Hospital, Rome, Italy; ^7^ Villa Sandra Institute, Rome, Italy; ^8^ Department of Psychology, Sapienza University of Rome, Rome, Italy; ^9^ Department of Biomedical, Surgical and Dental Sciences, University ‘La Statale’, Milan, Italy; ^10^ IRCCS Istituto Ortopedico Galeazzi, Milan, Italy; ^11^ Department of Biotechnology and Life Sciences, University of Insubria, Varese, Italy; ^12^ Istituti Clinici Scientifici Maugeri IRCCS, Tradate, Italy

**Keywords:** artificial intelligence, machine learning, natural language processing, clinical medicine, rehabilitation, neural networks, information science, digital health

## Abstract

Medicine has become increasingly receptive to the use of artificial intelligence (AI). This overview of systematic reviews (SRs) aims to categorise current evidence about it and identify the current methodological state of the art in the field proposing a classification of AI model (CLASMOD-AI) to improve future reporting. PubMed/MEDLINE, Scopus, Cochrane library, EMBASE and Epistemonikos databases were screened by four blinded reviewers and all SRs that investigated AI tools in clinical medicine were included. 1923 articles were found, and of these, 360 articles were examined via the full-text and 161 SRs met the inclusion criteria. The search strategy, methodological, medical and risk of bias information were extracted. The CLASMOD-AI was based on input, model, data training, and performance metric of AI tools. A considerable increase in the number of SRs was observed in the last five years. The most covered field was oncology accounting for 13.9% of the SRs, with diagnosis as the predominant objective in 44.4% of the cases). The risk of bias was assessed in 49.1% of included SRs, yet only 39.2% of these used tools with specific items to assess AI metrics. This overview highlights the need for improved reporting on AI metrics, particularly regarding the training of AI models and dataset quality, as both are essential for a comprehensive quality assessment and for mitigating the risk of bias using specialized evaluation tools.

## 1 Introduction

The advent of big data is radically changing every aspect of our civilization, and the medical field is among those most affected, creating opportunities for more comprehensive, data-driven clinical decision-making (1). Managing this wide amount of data should be possible only if automated, and AI has the potential to revolutionise and improve the efficiency and efficacy of men in many fields, including medicine. Large datasets are regularly generated in healthcare services, and AI can potentially utilise those datasets to improve most aspects of clinical medicine. Clinical medicine is the branch of medicine that focuses on the diagnosis, treatment, and care of patients. It involves the application of medical knowledge and skills to address the health needs of individuals (2). AI can be used for any step of clinical medicine: diagnosis, prognosis, clinical decision-making, treatment, patient education and even follow-up. Furthermore, thanks to its ability to analyse vast amounts of data, it could provide concrete help in applying the principles of personalised medicine, assisting the clinician in tailoring the best treatment specifically designed for each patient (3). On the other hand, while AI has the potential to revolutionise healthcare, its integration into clinical practice is still in the early stages (4). There is no clear quantification of the benefits of implementing AI-assisted tools in clinical medicine (5). AI can be a powerful tool, but, like any tool, clinicians need to be educated and trained in its use. Additionally, its limitations must be well-understood and carefully considered. For instance, AI inherently carries a risk of bias. These biases often stem from the data used to train AI models, which may reflect existing inequalities and inaccuracies in healthcare data (6). To mitigate these risks, standards for data diversity and continuous bias monitoring should be established (7, 8). The growing and enormous interest in the application of AI in medicine inevitably goes in two directions: evaluating the already existing applications in medicine and evaluating the possible role of future AI in every field of clinical medicine (9). We are still far from fully understanding and using AI to its full potential. A necessary step to reach this goal, which can change clinical medicine as we know it, is to have a clear picture of what is already done and understand as much as possible what is already established, what is working, and which research gaps are present. Furthermore, it is important to understand if current evidence reporting is sufficient to support researchers in building up from the present foundations. The number of guidelines for reporting studies on AI is increasing, a necessary step to accelerate knowledge growth in this area (10). Beyond reporting guidelines, reviewers have already made choices in different areas of clinical medicine that must be recognized to guide future research. We aim to produce a living systematic review of the AI applications clinically useful in rehabilitation, in collaboration and with the methodological supervision of Cochrane Rehabilitation according to their previous large experience with the rapid living systematic reviews on rehabilitation for COVID-19 (11). Creating a comprehensive map of the current understanding of AI applications in clinical medicine will allow us to develop a proper methodology and framework for comparison.

An overview of reviews provides a comprehensive synthesis of existing systematic reviews, offering a broad perspective on a topic, identifying research gaps, and highlighting key findings to inform future studies and decision-making (12). For this reason, an overview of reviews on the use of AI in clinical medicine allows us to categorise current evidence about it and identify the current methodological state of the art in the field. On one side, we want to see where and how it has mostly been used, providing a complete overview of the current literature; on the other, we want to describe the methodological tools used.

Thus, the primary aim of our overview of reviews is to verify the quality of reporting in SRs, to provide indications on qualitative and quantitative evaluation of AI to increase homogeneity and systematicity of future SRs and primary studies. In this way, we want to help future authors of secondary synthesis of the literature to build upon previous experiences; on the other, we want to contribute to the debate on how to better perform systematic reviews in the rapidly growing field of AI use in clinical medicine.

To achieve this, considering the specific characteristics of AI, a tailored evaluation tool for reporting SRs in the AI field is needed. An evolution of the PRISMA reporting guidelines, called PRISMA-AI, is currently under development, but it is not yet ready for use (13). Therefore, it seems necessary to employ interim tools. Thus, a secondary goal of our work is to propose a fast and effective tool that can bridge the temporary gap while waiting for PRISMA-AI.

## 2 Methods

### 2.1 Search strategy

The reporting of the overview followed the Preferred Reporting Items for Overviews of Reviews (PRIOR) Guidelines (14). The PubMed/MEDLINE, Scopus, Cochrane Library, EMBASE and Epistemonikos databases were screened from inception until 1 January 2024. Mixed, MeSH-terms and free-terms were used to perform the research. We used terms including artificial intelligence (i.e., “artificial intelligence”, ‘machine learning’, ‘deep learning’) applied in the field of clinical medicine (‘clinical medicine’). The search strategy was limited to systematic reviews and reviews (the complete search strategy is available in appendix A of the Supplementary Materials). The protocol was registered on the SFO database (https://doi.org/10.17605/OSF.IO/USB5J) (15). We included all systematic reviews, scoping reviews, and reviews with a structured search strategy that investigated the use of artificial intelligence in clinical medicine. Inclusion criteria were: (i) Systematic review, review, and scoping review with a structured search strategy; (ii) Investigation about the use of artificial intelligence or subgroups of AI; (iii) Application of AI tools in the field of clinical medicine, defined as reported in the introduction; (iv) English language; (v) Articles published in a peer-reviewed journal. Expert and narrative reviews and preprint manuscripts were excluded. All results were uploaded to Rayyan, a digital database for systematic reviews (16). Four blinded reviewers (AMC, LDA, AB, and RC) screened the results via title and abstract. After screening, in case of disagreement, the conflict was resolved with the support of two additional reviewers (FN, and GM). With the same procedure, the four reviewers analysed the full texts, and the conflicts were resolved with the intervention of the same two additional reviewers. The full texts were uploaded in PDF format (completed with Supplementary Materials) in a shared online folder.

### 2.2 Data extraction

The information of each included study was extracted and compared from two reviewers in a synoptic table on a sheet. In case of disagreement, a third author was involved. The data extracted were divided into qualitative information and AI classification (Complete data extraction strategy is reported in Table 1). Qualitative information extracted included the first author's name, the year of publication, the country, the number of databases consulted, the studies included, the research period, the field of medicine and studied clinical processes. Moreover, methodological information on using PRISMA/PRIMA-ScR checklists and the risk of bias (ROB) tools utilised were also collected, both the generic ROB tools and those specific to AI studies.

**Table 1:** Data extraction strategy.

Data classification	Data category	Type of data
Qualitative data	*Article information*	Author (Nom)
		Year of publication (Num)
		Country^a^ (Nom)
	*Search strategy*	Number of databases screened (Num)
		Years of research^b^ (Num/Nom)
		Number of studies included (Num)
	*Methodological approach*	Reporting according to PRISMA/PRISMA-ScR guidelines (Bin)
		Assessment of risk of bias (Bin)
		Risk of bias tool used (Nom)
	*Medical information*	Field of clinical medicine (i.e., cardiology, neurology) (Nom)
		Clinical processes involved (i.e., diagnosis, treatment) (Nom)
CLASMOD-AI	*Type of classification*	Item 1*:* Classification of data used as input (Bin)
		Item 2: Classification of AI model (i.e., ML, DL) (Bin)
		Item 3: Classification of AI training (i.e., supervised, unsupervised) (Bin)
		Item 4: Classification of metrics used to evaluate the model performance (i.e., sensitivity, AUROC) (Bin)

Abbreviation: AI, artificial intelligence; ML, machine learning; DL, deep learning; AUROC, area under the receiving operating curve; Nom, nominal variable; Num, numerical variable; Bin, binary variable. ^a^Country of first Author. ^b^The variables were considered nominal for research from inception, and numeral for a specified time window reported in years.

#### 2.2.1 Classification model in reporting artificial intelligence metrics in the systematic review (CLASMOD-AI)

Lastly, we implemented a tool to evaluate the quality of reporting in SRs on AI (CLASMOD-AI). The tool aims to understand the classificatory categories used by systematic reviewers to group primary studies. Such a tool could be a reference for future authors when choosing how to classify AI studies in their reviews and will serve our scope within the field of rehabilitation. Starting from reporting tools already developed for other types of research studies involving AI (i.e., CONSORT-AI (17) and SPIRIT-AI (18), APPRAISE-AI (19) and TRIPOD-AI (20)), we analysed and extrapolated the key characteristics. An open discussion with the Board of the Italian Society of Artificial Intelligence in Medicine (SIIAM) produced the final proposal, which was purposefully limited to the most important items. The tool is not yet validated, but it is deemed necessary given the current lack of appropriate instruments. According to the current version of the tool CLASMOD-AI, we evaluated if the following classificatory items were reported in the SRs: (i) type of data (Text, Images, etc.), (ii) AI models (Machine Learning, Deep Learning), (iii) model training (Supervised, Unsupervised, Reinforcement), (iv) model performance metrics (i.e., Sensitivity, Specificity).

## 3 Results

**Figure 1:**
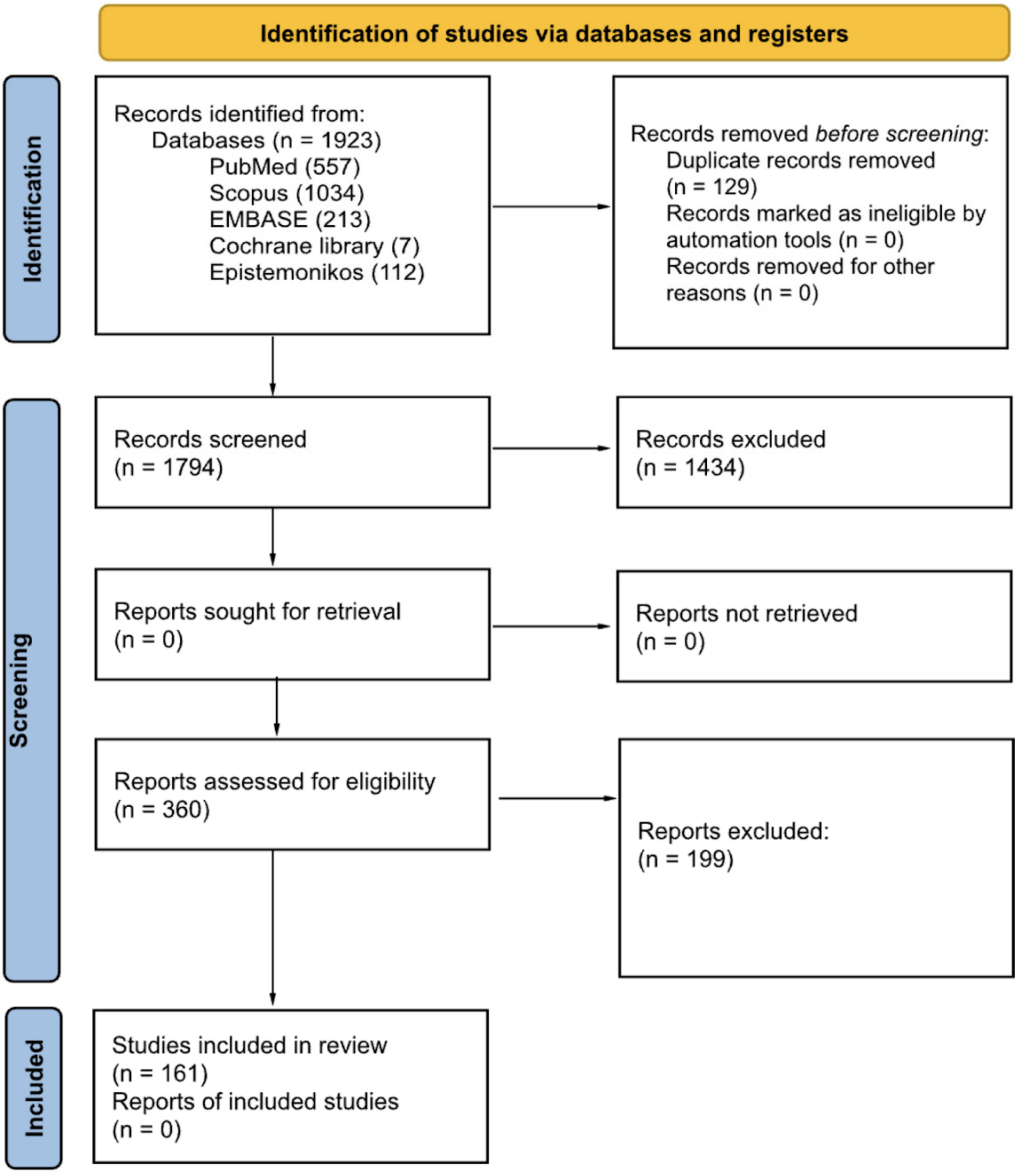
Flow-diagram of inclusion process.

After full-text screening, 161 reviews (The complete list is available in Appendix B of the Supplementary Materials) (151 systematic reviews and 10 scoping reviews) were included in the final synthesis (The search process is reported in Figure 1).

### 3.1 Article information

**Figure 2:**
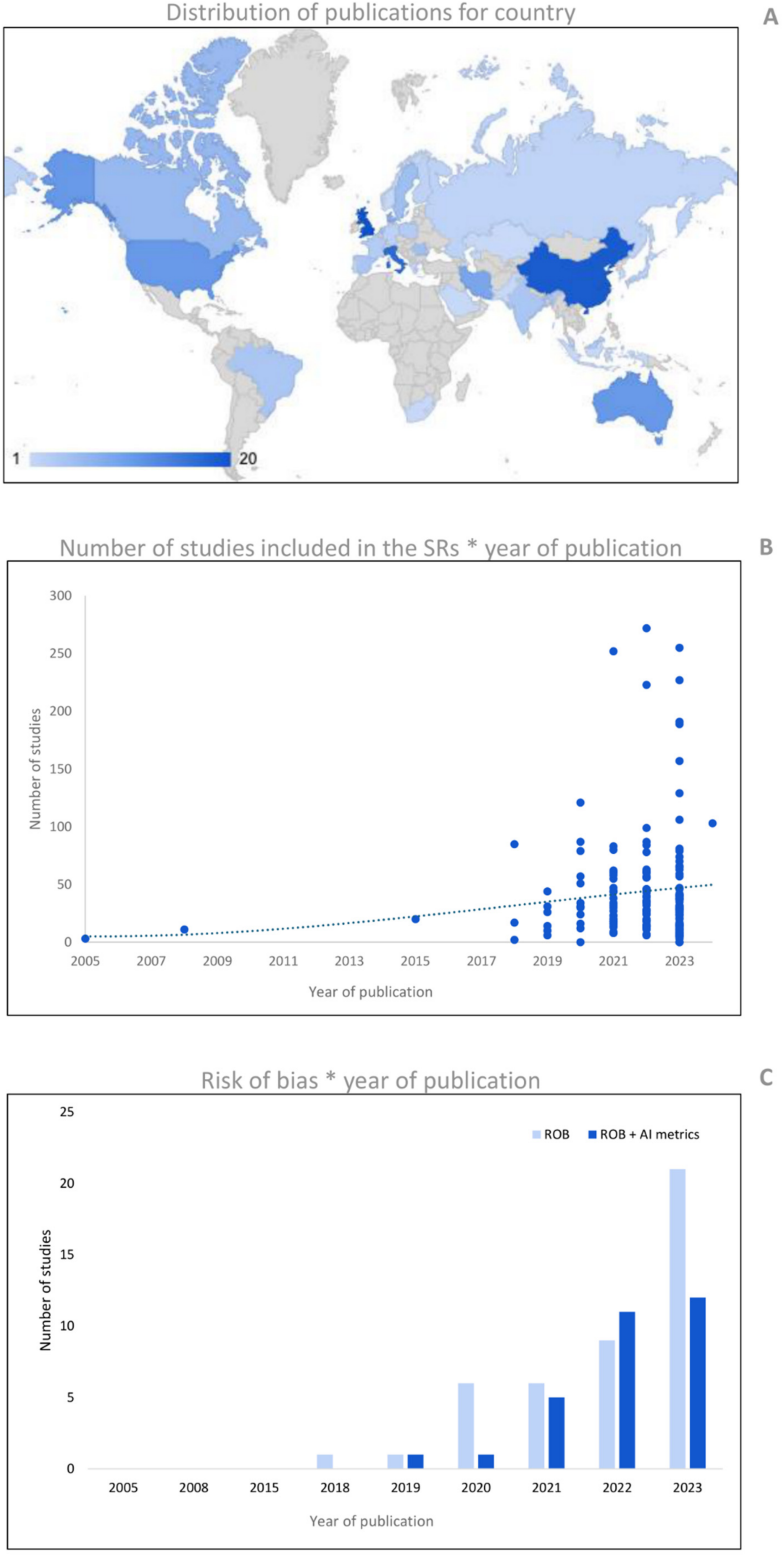
**(A)** Shows the number of publications per country (based on the affiliation of the first author). **(B)** Reports the number of publications of AI reviews for the year of publication. The cubic polynomial trendline shows the sprout of primary studies included in the reviews, specifically during the last five years. Panel C shows the number of SRs that performed a risk of bias (ROB) assessment for years of publication. The ROB tools were computed as ‘ROB’ for conventional methodological quality assessment, and ‘ROB + AI metrics’ for ROBs that evaluate specifically the AI metrics.

The publication date of the included studies ranged from April 2005 to January 2024, with an average increase in the last three years of 10.4 ± 2% for each year. The United Kingdom (12.4%), China (11.8%), and Italy (10.6%) are the countries most represented in terms of the total number of reviews published (Figure 2A).

### 3.2 Search strategy

The average (± standard deviation) number of databases screened for the research was 3.7 ± 1.7. The research was conducted since inception in 43.5% of SRs, for a predefined time window in 52.2% of SRs, and in the remaining 4.3%, the information was missing. In the SRs that performed the research for predefined time windows, 14.3% searched for at least five years, 32.1% from 6 to 10 years, 23.8% from 11 to 20 years, 26.2% from 21 to 30, and 3.6% over 30 years, with an average of 13.2 ± 8.4 years. The average number of primary studies included was 45.1 ± 48.7. Comparing the number of primary studies included based on their year of publication, shows an increase, particularly over the last five years (Figure 2B). Except for one, all reviews reported the number of primary studies included in the synthesis, and in total, 7,672 studies were included.

### 3.3 Methodological approach

Seventy-seven percent of the studies reported according to the PRISMA (or PRISMA-ScR) guidelines and 49.1% of reviews performed a risk of bias (ROB) analysis of included studies. We found four approaches in the ROB analysis: (i) 62.0% of these reviews used a ROB tool for the assessment of methodological quality without specific items for AI metrics; (ii) 16.5% used only a tool with specific designed items for the assessment of quality of AI metrics; (iii) 13.9% used both approaches combining two different tools for the assessment of methodological quality and quality of AI metrics; and finally (iv) 7.6% used a single tool that combined items to assess both general methodological quality and quality of AI metrics. In general, 39.2% of the reviews analysed the quality of AI metrics alone or in combination with other tools. An increase in the use of ROB tools for assessing methodological quality was observed in recent years. However, concerning the use of dedicated tools for AI (or with specific items for AI), the increase is smaller and more recent (see Figure 2C). The most used ROB tool was the QUADAS-2 (41.8%), followed by the prediction model risk of bias assessment tool (PROBAST) (11.4%), and the radiomics quality score (RQS) (10.1%). The complete percentages of tools used for ROB assessment are reported in Table 2.

**Table 2:** Description of risk of bias tools.

Tool	*N* of items	Domains/sections	Items for AI metrics assessment	Scope of the tool	Percentage of use
QUADAS-2	7	1. Patient selection, 2. Index test, 3. Reference standard, 4. Flow and timing.	None	Assessment of ROB and applicability of primary diagnostic accuracy studies.	41.8%
PROBAST	20	1. Participants, 2. Predictors, 3. Outcome, 4. Analysis.	This tool has a specific domain (analysis) where there are nine questions to evaluate the use of predictors, to estimate the probability that a condition or disease is already present (diagnostic model) or will occur in the future (prognostic model) or updating (for example, extending) prediction models, both diagnostic and prognostic.	Assessment of the quality, ROB and applicability of prediction models.	11.4%
RQS	16	1. Image protocol, 2. Radiomics features extraction, 3. Data analysis and statistics, 4. Model validation, 5. Clinical validity, 6. Pen science.	Six domains with sixteen questions for analysis for the quality of intelligence (AI) and radiomics papers; a maximum score of 36 represents as an indicator of superlative quality.	Evaluate the methodological quality of radiomics-based investigations, identifying high-quality results as well as issues limiting their value and applicability.	10.1%
JBI	11	None	None	A checklist to analyse SRs about the methods utilised to synthesise the effectiveness or otherwise of a practice, with the judgement of a series of complex steps and different types of evidence.	7.6%
CHARMS	35	1. Source of data, 2. Participants, 3. Outcome(s) to be predicted, 4. Candidate predictors (or index tests), 5. Sample size, 6. Missing data, 7. Model development, 8. Model performance, 9. Model, evaluation, 10. Results, 11. Interpretation and discussion.	Four specific domains with a total of twelve questions that analyse the evaluation, development and performance of the models used.	To help form a review question for and appraisal of all types of primary prediction modelling studies, including, regressions, neural network, genetic programming, and vector machine learning models.	7.6%
Rob2		1. Bias arising from the randomization process, 2. Bias due to deviation from intended interventions, 3. Bias due to missing outcome data, 4. Bias in measurement of the outcome, 5. Bias in selection of the reported results.		Cochrane risk-of-bias tool for randomised trials to assess the risk of bias in randomised trials and crossover trials.	5.1%
CLAIM	21	1. Title, 2. Abstract, 3. Introduction, 4. Methods		Is a tool to promote complete and consistent reporting of AI science in medical imaging and has been adopted widely in several medical specialties that involve imaging and AI.	3.8%
TRIPOD	22	1. Title, 2. Abstract, 3. Introduction, 4. Methods, 5. Results, 6. Discussion. 7. (other information)	This evaluation tool has specific questions into all domains; the questions analyse the developmental and potential of the prediction models used; there are also specific questions about the type of variables and the interpretation of the results.	To harmonise the landscape of prediction model studies and to provide transparent reporting of studies developing, validating, or updating a prediction model regardless of whether regression models or machine learning methods have been used, whether the model are used for for diagnostic, prognostic, monitoring or screening purposes, irrespective of the medical domain and of the outcomes predicted or predictors being used.	2.5%
NOS	8	1. Selection, 2. Comparability, 3. Exposure/outcome.	None	To assess the quality of nonrandomised studies with its design, content and ease of use directed to the task of incorporating the quality assessments in the interpretation of meta-analytic results.	2.5%
Other	–	–	–	–	7.6%

Abbreviations: QUADAS-2, quality assessment of diagnostic accuracy studies v. 2.0; PROBAST, prediction model risk of bias assessment tool; RQS, radiomic quality score; JBI, the Joanna Briggs Institute Critical Appraisal Tools Checklist for Systematic Reviews and Research Syntheses; CHARMS, CHecklist for critical appraisal and data extraction for systematic reviews of prediction modelling studies; Rob2, a revised cochrane risk-of-bias tool for randomised trials; CLAIM, checklist for artificial intelligence in medical imaging; TRIPOD, the transparent reporting of multivariable prediction models for individual prognosis or diagnosis; NOS, The Newcastle-Ottawa Scale.

### 3.4 Medical information

**Figure 3:**
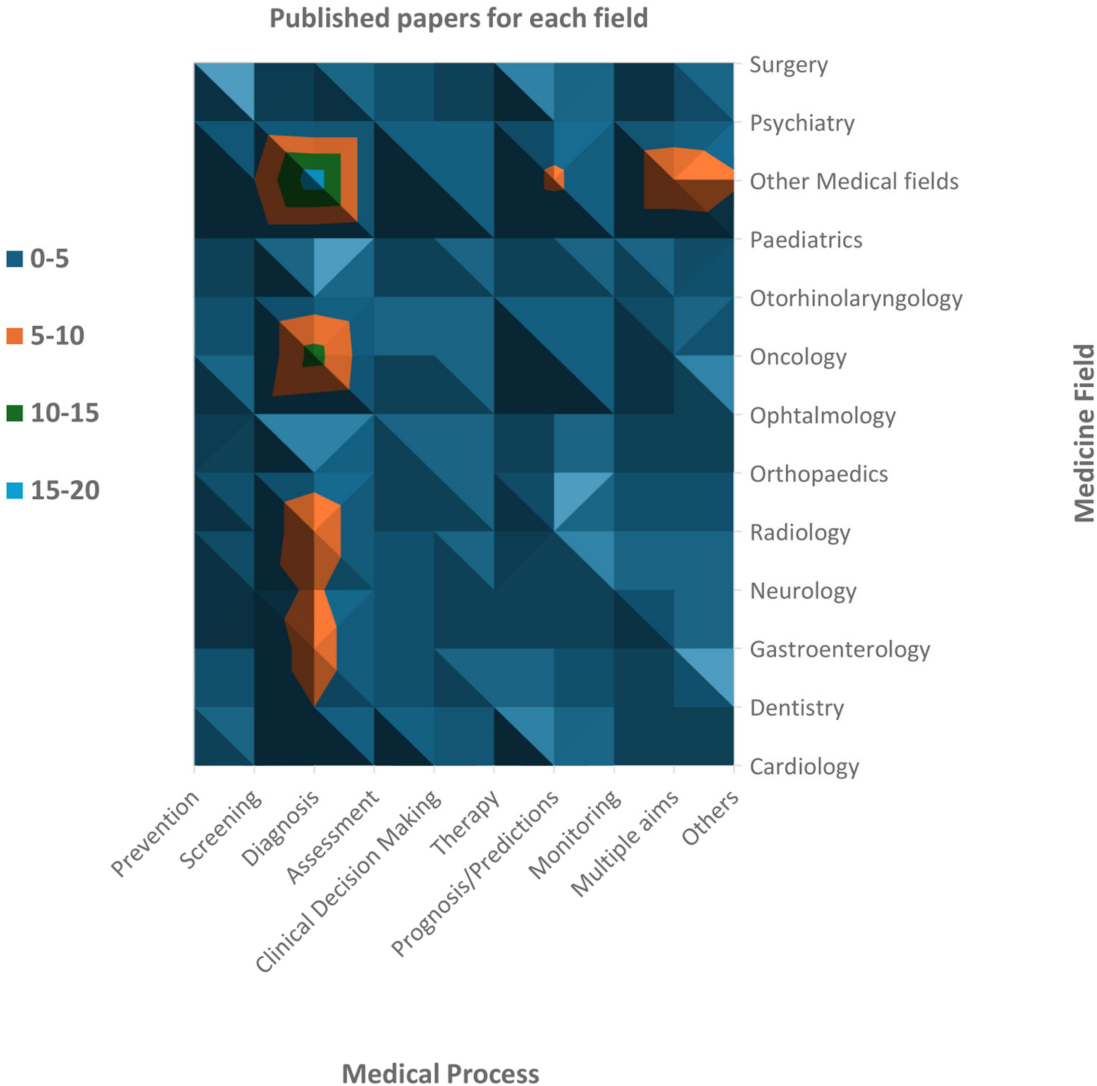
Number of revised papers for each medical field and each phase of the medical process. Oncology is the medical field most investigated, followed by neurology and radiology (including neuroradiology). Dentistry, gastroenterology and orthopaedics have also been widely studied. Among the medical processes, diagnosis is the most frequent scope of AI analyses.

We found SRs in 41 fields of clinical medicine, and the most frequent were oncology (13.9%), neurology (7.9%), radiology (7.9%), gastroenterology (6.0%), and dentistry (5.3%). We found that AI was used mostly for diagnosis (44.4%), prognosis/predictions (13.9%), and screening (9.3%) purposes. The oncology field used AI principally with the scope of diagnosis (57.1%) and prognosis/prediction (23.8%). The choice of AI models is primarily driven by the specific objective (i.e., diagnosis and prognosis/predictions) rather than the medical field itself. For instance, Convolutional Neural Networks (CNN) and Support Vector Machine (SVM) are frequently used across a wide range of fields, with algorithms like Random Forest (RF), Least Absolute Shrinkage and Selection Operator (LASSO), Deep Neural Network (DNN), and Logistic Regression (LR) often complementing them to enhance diagnostic accuracy and clinical decision-making. This widespread usage stems from the proven effectiveness of these algorithms in diagnostic tasks across diverse medical domains. In summary, these algorithms were used often in tandem to provide more accurate, efficient, and scalable solutions for medical diagnosis, enhancing the precision of disease detection and supporting clinical decision-making across a variety of healthcare applications. Medical fields with a low prevalence in our sample were collapsed into ‘other medical fields’, but even in this case, the main objective of using AI remained diagnosis (35.3%). Furthermore, 13.2% of reviews investigated using AI for multiple purposes or with specific aims that were not yet categorizable (Figure 3).

### 3.5 CLASMOD-AI

The most frequently reported information about AI metrics across the SRs was the category of AI models employed (84.5%). In contrast, there was a notable lack of reporting on model training categories, which was present in only 25.6% of cases. Only 15.5% of the SRs met all four items of the CLASMOD-AI, while 5.6% did not meet any item (Table 3). In SRs that satisfied just one item of CLASMOD-AI, 58.3% reported the AI model classification, while none reported the classification according to AI training. In SRs that satisfied two items, the most common combinations were items 2 and 4 (47.1%) and items 1 and 2 (33.3%), with no studies reporting any other combinations. Finally, in SRs that met three items, the most common combination was items 1, 2, and 4 (76.9%). In conclusion, item 3 (classification according to AI training) was generally the least reported, with a significant lack of information regarding AI training (e.g., supervised, unsupervised, generative AI). Moreover, the evaluation of the datasets was not conducted using dedicated measures such as ML-DQA, ROBUST-ML, MINIMAR, or the V7 Tool. These methodologies and tools are specifically designed to assess the quality, robustness, and reliability of AI datasets, ensuring that they meet predefined standards for accuracy, completeness, and bias mitigation. However, more than half of the SRs (63.3%) provide information on the dataset volume, either in terms of the number of acquisitions or the number of patients included.

**Table 3:** Results of CLASMOD-AI.

Classification name	Items	% (yes)
*CLASMOD- AI*	1. Classification of data used as input	62.9
	2. Category of AI models employed	84.5
	3. Category of model training	25.6
	4. Classification of metrics used to evaluate the model performance	68.1
	No items	5.6
	At least 1 item	14.9
	At least 2 items	31.7
	At least 3 items	32.3
	All items	15.5

## 4 Discussion

This overview of reviews aims to create a synthetic portrait of the use of AI in clinical medicine, merging the results of systematic reviews with a methodological focus on the reporting, risk of bias, and classification of AI tools used. In recent years, primary studies on AI in medicine have increased exponentially. SRs allow us to summarise on a large scale the major characteristics of the different fields of clinical medicine and the medical processes involved (i.e., diagnosis, prognosis, clinical decision-making and treatment). To our knowledge, this is the first overview of SRs on this emerging topic. The choice to use the overview of SRs design, despite some limitations discussed in the limitations section, provided us with a wider view on the current evidence regarding the clinical use of AI in medicine. This approach also made it possible to extract methodological features and related information reporting.

We found 161 SRs, including about 7,500 primary studies, that investigate the use of AI for medical purposes in a large range of clinical medicine fields, most of which were published in the last five years. This finding confirms that AI is becoming more integrated into clinical medicine, as reflected by the broad range of medical fields covered in the included SRs (41 fields). This accounts for the phenomenon's complexity and the sprout of using AI in clinical medicine and other non-medical disciplines. The use of AI is more studied in some medical areas, such as oncology, neurology and radiology, but also in gastroenterology and dentistry.

Despite the incredible proliferation of studies on the clinical use of AI, as evidenced by the works included in the reviews considered (7,672 studies), the overall methodological quality is disappointing. CLASMOD-AI, our newly developed tool for screening the quality of reporting in AI-related SRs, allowed us to highlight important issues in the included studies. The significance of CLASMOD-AI lies in the fact that, currently, there is no established guidance or tool available to help authors classify AI studies in SRs (for more information, see the appendix D of the Supplementary Materials). While PRISMA-AI guidelines are under development and will offer comprehensive reporting standards for SRs involving AI in healthcare (13), CLASMOD-AI addresses the immediate need for a practical, simplified framework. It serves as a valuable interim solution, offering a structured approach to address the challenges posed by the heterogeneity of AI studies in SRs.

An important challenge in synthesising evidence from primary studies on AI in clinical medicine is their heterogeneity. To prevent confusion among readers, it is essential for every systematic review to clearly specify any specific constraints in the study's inclusion criteria. These constraints could relate to the type of input data, the category and type of AI models used, or the evaluation metrics considered. If no constraints limit the selection of studies to a particular instance within these categories (e.g., focusing only on studies using images as data), then these categories can serve as classification criteria to group the primary studies for clearer analysis and reporting. In our analysis, many authors opt to categorise studies based on the type of AI models employed, with approximately 85% of the SRs distinguishing primarily between machine learning and deep learning models. In contrast, only about one-quarter of these studies utilise the training category of AI models as a classification criterion. This is likely because most AI models in clinical medicine rely on supervised learning. Since supervised learning represents a single category, it often cannot be used as a classification criterion. Furthermore, the data type is a source of high heterogeneity among the primary studies, and it is often employed to group studies (around 63% of the SRs). Lastly, employing model evaluation metrics as criterion for grouping studies is crucial for quantitatively synthesising results and conducting meta-analyses (around 68% of the SRs).

The classification patterns observed in our analysis highlight important considerations for future reviews. Researchers should consider the dominant classification schemes and their implications for synthesis and reporting. Specifically, distinguishing studies based on data types or evaluation metrics can provide more structured and insightful analyses. Ultimately, this approach will aid in harmonising evidence and improving the comprehensiveness of reviews in the evolving field of AI in clinical medicine.

Furthermore, nearly a quarter of the studies did not report following PRISMA guidelines, and less than 50% conducted a risk of bias (ROB) analysis of the included studies. Naturally, the lack of use of these tools raises significant concerns about their quality (21). Furthermore, AI is a complex subject and also different and more technological from other issues we are used to studying in health care, which introduces additional methodological challenges. Many biases are particularly relevant or specific to AI studies, including inappropriate train-test split, lack of diversity within the input data, historical bias, representation bias, evaluation bias, aggregation bias, population bias and sampling bias (22). To address these challenges in evaluating the quality of AI studies, an adaptation of generic ROB tools to incorporate specific items for AI studies is required. AI-specific extensions and reporting guidelines have been created for randomised control trials (RCTs) in 2020 CONSORT-AI (Consolidated Standards of Reporting Trials–AI) (17) and SPIRIT-AI (Standard Protocol Items: Recommendations for Interventional Trials-AI) (18) were published. However, only 19% of RCTs in the AI medical field published after the start of 2021 cited the CONSORT-AI guidelines (23). This highlights possible difficulties and delays in the adoption of these tools. To support this evidence, we identified only one SR reporting the use of CONSORT-AI in ophthalmology (24). Moreover, RCTs represent only a minority of primary studies in the medical AI field and extensions of ROB tools and reporting guidelines for other categories of AI studies were released very recently, such as APPRAISE-AI (19) and TRIPOD-AI20 for clinical decision support and prediction models in 2023–2024, or still under development, such as QUADAS-AI for diagnostic studies (25) and PROBAST-AI for prediction models (26), both announced in 2021. Given that over 50% of the reviews in our study that assessed the risk of bias used QUADAS-2 or PROBAST, it is crucial to expedite the release of the anticipated AI extensions of these tools. Considering potential delays in their adoption and the rapid pace of scientific production in this field, there is a risk that a growing number of SRs on AI across various medical fields may inadequately assess study quality. This could result in an expanding body of incomplete or potentially misleading evidence.

Two different checklists developed specifically for imaging studies, RQS (27) and CLAIM (28), were widely used in reviews focusing on radiology. This alternative approach creating quality assessment tools tailored to the type of data rather than the study design could prove promising if similar tools are developed for text-based or structured data in the future.

Moreover, the lack of dedicated dataset evaluations (29–31) suggests that potential data inconsistencies, biases, or other quality concerns may not have been systematically identified or addressed. In addition to the performance of the algorithms, it is crucial to understand certain characteristics of the datasets, as this information is essential for assessing their quality and, consequently, evaluating the quality of the AI model. As a result, any conclusions drawn from these datasets should be approached with caution, as underlying data quality issues could affect the overall performance of AI models.

Regarding the medical processes, diagnosis is the most important purpose independently represented by the clinical medical fields. AI has demonstrated high accuracy and diagnostic potential across various areas, including early detection of cancers (32–34) and osteoarthritis (35), and predicting disease progression in conditions like Parkinson's (36) and otitis media (37). Radiomics, AI-enhanced imaging, and machine learning algorithms have improved diagnostic processes, with promising results in personalized care and tumor detection. However, while AI models show competitive performance compared to human experts in detecting specific conditions (e.g., prostate cancer, coeliac disease, and hip fractures) (34, 38, 39), challenges remain in their widespread clinical adoption. These include the need for more robust, diverse datasets and the resolution of methodological issues that impact model generalizability.

However, AI is influencing all domains, including those where human judgement is typically expected to be decisive, such as therapy choice and clinical decision-making. Despite these advancements, their application is still in the early stages, showing promising results but facing significant challenges. In decision-making, AI algorithms enhance prediction accuracy, risk classification, and disease progression tracking. For instance, AI has shown higher sensitivity in lung nodule assessment (40), detecting vertical root fractures in endodontics (41), and clinical decision support for the hospital setting (42), often outperforming traditional methods. These models provide clinicians with data-driven insights that support dynamic care strategies, adaptive treatments, and precision medicine. AI is also applied in therapeutic support in areas like pediatric anesthesia (43), stroke treatment (44), and knee arthroplasty (45), aiding surgical planning, postoperative monitoring, and outcome prediction through deep learning models.

It is worth noting that this last aspect will be promising, for example, in patients with multiple conditions, mitigating the problems of traditional clinical medicine as the hyperspecialization with a lack of integrated competencies. In this context, intelligent clinical decision support systems should be able to manage complexity and minimise iatrogenic risks (46).

In medical education, AI allows for personalized, scalable learning, and it improves healthcare management by supporting decision systems for chronic disease and multimorbidity. Additionally, AI automates neonatal pain assessment (47), coordinates clinical nutrition research (48) for more personalized care, and optimizes outcomes in fields like ophthalmology and surgery. Furthermore, it contributes to public health by predicting and managing chronic disease outcomes, enhancing healthcare efficiency across diverse non-diagnostic areas.

The SRs regarding AI in clinical medicine conducted in recent years have thus primarily focused on diagnosis, screening, and prognosis in oncology and radiology. The numbers are striking: despite identifying reviews across 41 different fields of clinical medicine, oncology, and radiology together account for ∼22% of the total, while studies on diagnosis, screening, and prognosis cover over 65%. AI can help bring order to complex decisions based on images or molecular markers using CNN, SVM, LASSO, RF, DNN and LR. However, we believe it is limiting to consider AI merely as a diagnostic tool. As abovementioned, AI can potentially be successfully employed in other processes, such as clinical decision-making (49), therapeutic applications (50), or patient education (51). In these domains of clinical medicine, research appears to still be in its early stages, and the potential of AI is largely untapped. We believe that our work can incentivise clinical researchers to identify new and exciting AI-based clinical approaches.

The number of publications has increased, particularly since 2018, when we witnessed exponential growth. We can also see how, over the years, there has been increasing attention in SRs to using quality tools specifically built for evaluating medical research studies involving AI (26, 52).

Despite ChatGPT emerging as a leader among AI systems utilising large language models (LLMs), it is important to note that we found no reviews investigating its use in clinical medicine despite including over 160 reviews. This is likely because, by its very nature, it is more suitable for use in healthcare for purposes other than clinical applications, such as education, research, or improving administrative processes (53). Another possible reason for the absence of SRs on LLMs is the time required to accumulate sufficient primary studies on the topic. Since LLMs are relatively new, there is a natural delay before enough research is available to conduct comprehensive reviews on specific areas of clinical medicine.

Regarding geographical distribution, England, China, and Italy are the countries with the highest SR production. However, we found SRs published from all the major continents (Africa, the Americas, Asia, Australia, and Europe). The ubiquitous publication of studies is a potential positive indicator of AI diffusion worldwide. Although during the last 30 years, medical inequity in human resources for health was reduced, all-cause mortality was relatively higher in countries and territories with a limited health workforce (54). As declared by the WHO, it is important to develop equity-oriented health workforce policies, expanding health financing to achieve universal health coverage by 2030 (55). Undoubtedly, AI will play a fundamental role in this scenario. It is important to monitor these geographical aspects because, a great impact of AI on healthcare systems worldwide might create a unique opportunity to reduce inequity or pose a possible risk to augment the gap between high-income countries and the global south.

### 4.1 Ethical consideration and iniquities

Ethical considerations are essential to ensure that AI does not harm the patient and caregivers and that its application ensures a medical-social benefit. Developers economically interested in the development of AI may have prejudices for example about gender, origin, and the motor-cognitive functioning of the person. It is essential to create tools that also correctly analyze ethical aspects at every stage such as for development, validation, implementation, and surveillance of the AI Application in Healthcare. To date, SPIRIT-AI, for example, contemplates items related to ethics (56).

While artificial intelligence represents a real possibility of improving the well-being and health of all people in every country, there is a real risk that it could increase the inequities between developed and developing countries and among and between different social classes of the same nations.

In fact, artificial intelligence is part of a broader context of Digital Health that is supported by other technology domains that are considered as social determinants of health, such as big data analytics, the internet of things (IoT), next-generation networks (e.g., 5G), and privacy-preserving platforms, e.g., blockchain. The development and the healthcare use of these other technological domains is certainly not homogeneous worldwide (57).

### 4.2 Limitations and strengths

Our work is not without limitations. The use of an overview enables us to adopt a global approach to a broad topic, such as the application of AI in clinical medicine. However, this tool has an inherent limitation: it is more temporally delayed compared to a traditional systematic review, precisely because it involves secondary analysis. The topic considered is extremely broad. We aimed to come as close as possible to identifying all studies on the subject, as demonstrated by the significant screening effort (1,923 reviews, of which 161 were included). Nevertheless, the breadth of the topic leaves the possibility that our search string may have missed some studies. Another limitation of our study is that we did not conduct an analysis of how many primary studies are duplicated within our overview. However, since the methodological focus was on data reporting, we do not consider this a significant limitation for our purposes. Furthermore, the CLASMOD-AI tool has not yet been validated in a dedicated research paper. On the other hand, it has been developed through a structured internal consensus process by SIIAM. The proposed ad interim tool (CLASMOD-AI) does not consider the ethical aspects of AI implementation and reporting in systematic reviews. For future use, the part relating to ethical considerations must be implemented and validated. Moreover, it needs to be considered that, to our knowledge, there is still no reliable tool available for quality assessment of the reporting in SRs, probably because AI is a very novel topic and the correct methodology for research and study reports is still under investigation. Another limitation of our study is that our data extraction process did not fully capture the depth of information in the SRs. Due to the large and heterogeneous number of SRs, our focus was primarily on general and methodological considerations. Future research could benefit from a more targeted examination of specific clinical domains to extract more meaningful insights into AI's state of the art within those areas from SRs. Finally, a possible overlap in the primary studies included in the SRs was not investigated. However, this limit may be mitigated by the large number of fields of clinical medicine found across SRs.

On the other hand, our overview has many strengths. To the best of our knowledge, it is the first study to conduct an overview of reviews on the use of AI in clinical medicine. We analysed over 160 reviews from a bibliographical, methodological, and content perspective and summarised the results. In our work, we introduced a quick and novel methodological assessment of the reporting in AI-related reviews, which future studies can use.

## 5 Conclusions

The methodological quality of the analysed studies is unsatisfactory, as only a few of the included reviews utilize AI-specific tools for ROB analysis and dataset evaluation. Given that dataset quality is a critical factor in AI training, this lack of clarity may lead to challenges in validating the AI models used in research studies. Our newly developed tool to assess the quality of reporting of SRs regarding AI, CLASMOD-AI, allowed us to discover that an unsatisfactory percentage of SRs reported critical elements of primary studies. AI in clinical medicine is currently used primarily for diagnosis (44.4% of the studies considered) in oncology and radiology. Many countries from all major continents have conducted studies related to AI, with England, China, and Italy being the most prominent contributors. Most studies have been published in the last five years, with a continuously increasing trend year after year. Despite the proliferation of studies on the topic, the potential for AI to improve the work of clinicians worldwide remains largely untapped.

Clinicians around the world must be aware of the potential risks associated with the use of AI: systematic reviews do not yet seem adequate to identify the ROB of primary studies effectively, ethical issues have not yet been resolved, and much research is still needed to identify all the domains and areas of medicine where AI can be effectively utilised.
